# Time to Regain Birthweight and Association with Neurodevelopmental Outcomes among Extremely Preterm Newborns

**DOI:** 10.21203/rs.3.rs-3249598/v1

**Published:** 2023-09-14

**Authors:** Gregory Valentine, Krystle Perez, Thomas Wood, Dennis Mayock, Janessa Law, Sarah Kolnik, Katie Strobel, Olivia Brandon, Bryan Comstock, Patrick Heagerty, Sandra Juul

**Affiliations:** University of Washington; University of Washington; University of Washington; University of Washington - Seattle Children’s Hospital; University of Washington; University of Washington; University of Washington

**Keywords:** Time to regain birthweight, weight loss, neonatology, prematurity, neurodevelopment

## Abstract

**Objective:**

Determine association between time to regain birthweight and 2-year neurodevelopment among extremely preterm (EP) newborns.

**Study Design:**

Secondary analysis of the Preterm Erythropoietin Neuroprotection Trial evaluating time to regain birthweight, time from birth to weight nadir, time from nadir to regain birthweight, and cumulative weight loss with 2-year corrected Bayley Scales of Infant and Toddler Development 3rd edition.

**Results:**

Among n = 654 EP neonates, those with shorter nadir-to-regain had lower cognitive scores (2–4 days versus ≥ 8 days: −3.5, [CI −7.0, 0.0]; ≤1 day versus ≥ 8 days: −5.0, [CI −10.2, 0.0]) in fully adjusted stepwise forward regression modeling. Increasingly cumulative weight loss was associated with lower cognitive scores (−50 to <−23 percent-days: −4.0, [95% CI −7.6, −0.4]) and language scores (≤−50 percent-days: −5.7, [CI −9.8, −1.6]; −50 to <−23 percent-days: −6.1, [CI −10.2, −2.0]).

**Conclusion:**

Faster nadir-to-regain and prolonged, severe weight loss are associated with adverse 2-year neurodevelopmental outcomes.

**Trial registration:**

PENUT Trial Registration: NCT01378273. https://clinicaltrials.gov/ct2/show/NCT01378273

## Introduction

Newborn providers often closely monitor the number of days after birth an infant takes to regain their birthweight (time-to-regain) due to its association with short- and long-term outcomes.^1–6^ However, there is limited evidence evaluating time-to-regain among preterm newborns and its effects on long-term neurodevelopmental outcomes, especially for those born extremely preterm (EP), before 28 weeks’ gestation.

EP newborns are at risk for severe weight loss after birth due to high insensible water losses and immature kidney tubular function, among other factors.^7–16^ Degree of weight loss is associated with time-to-regain, and those with minimal weight loss often regain birthweight earlier. Critically ill premature newborns are commonly provided high-volume fluid resuscitation that limit or prevent weight loss after birth and shorten time-to-regain. We and others have demonstrated that minimal weight loss from birthweight (< 5%) among EP newborns is associated with adverse in-hospital outcomes such as necrotizing enterocolitis.^16–18^ Thus, it is possible that a *rapid* time-to-regain is associated with adverse outcomes. These findings conflict with recently published recommendations defining time-to-regain more than 14 days as being associated with malnutrition, implying a shortened time-to-regain is optimal.^19^ These conflicting recommendations pose unanswered questions. Specifically, what is the optimal time-to-regain among EP newborns to optimize long-term neurodevelopmental outcomes?

**Figure 1:**
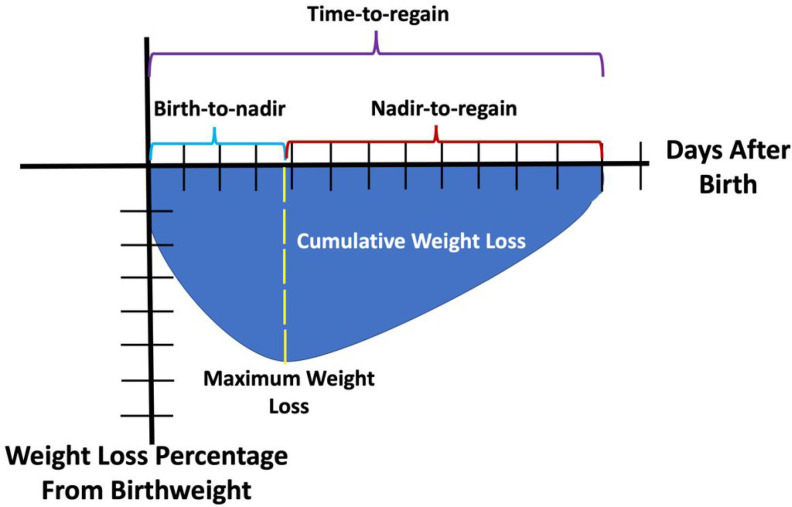
Time to regain birthweight and the associated components. Visual depiction of time to regain birthweight (time-to-regain) and its associated components including birth to nadir of weight loss (birth-to-nadir), nadir of weight loss to regain of birthweight (nadir-to-regain), maximum weight loss, and cumulative weight loss. Cumulative weight loss is the blue shaded area above the weight loss curve and is the sum of the percent weight loss for each day the weight was below birthweight. Maximum weight loss is the maximum percent weight loss from birthweight.

Time-to-regain is comprised of multiple components (Fig. 1): time from birth to weight loss nadir (birth-to-nadir), time from weight loss nadir to regain birthweight (nadir-to-regain), maximum weight loss percentage from birthweight, and a measure of degree of weight loss over time, or cumulative weight loss (calculated as the sum of the percent weight loss for each day the weight was below birthweight). Each of these components influence the overall time-to-regain, and it is unclear what, if any, roles these factors have on neurodevelopmental outcomes. To examine the association of time-to-regain on outcomes, one must consider each component that impacts the overall time-to-regain. We performed a post-hoc, secondary analysis of the Preterm Erythropoietin (Epo) Neuroprotection Trial (PENUT)^20^ to evaluate associations between time-to-regain, birth-to-nadir, nadir-to-regain, maximum weight loss percentage, and cumulative weight loss with neurodevelopmental outcomes at 2-years corrected age (CA). We hypothesized that *rapid* time-to-regain was associated with lower neurodevelopmental scores at 2-years CA.

## Subjects and Methods

PENUT was a double-blinded, randomized, multi-center, placebo-controlled trial of EP newborns, defined as 24w0d-27w6d at birth, evaluating Epo administration compared to placebo on neurodevelopmental outcomes.^20^ Details of the study protocol and primary outcomes have been previously published.^20^ All study subjects’ legal guardians provided informed consent for their newborns to be included in PENUT. Institutional Review Board approval was obtained at each site.

We conducted a secondary analysis of PENUT infants who survived to 2-years CA and underwent Bayley Scales of Infant and Toddler Development 3rd edition (Bayley-III) assessment. Daily fluid and weight data over the first 14 days were collected as part of the PENUT protocol.

### Statistical Analyses

Associations between time-to-regain, including its four components, and each of the Bayley-III subscales were first explored descriptively using locally estimated scatter plot smoothing (loess) curves. As weight data were not available beyond day 14, for visual representation and calculation of time-to-regain, any infant who had not regained birthweight by day 14 was assumed to have regained birthweight on day 15. We then determined quartiles of time-to-regain for further analyses (see Results).

For all inferential analyses, generalized estimating equations (GEE) with robust standard errors were used to appropriately account for potential correlation of outcomes for same-birth siblings.^21^ Baseline and demographic factors were compared across quartiles of time-to-regain using a multivariate Wald test. Fluid administration over the first 14 days was averaged relative to birthweight to provide a value of mL/kg birthweight/day.

Using GEE linear regression models adjusting for gestational age (GA) and Epo treatment group, we examined the association between neurodevelopmental outcomes at 2-years CA and maternal and infant factors, time-to-regain, birth-to-nadir, nadir-to-regain, maximum weight loss, or cumulative weight loss quartile. Infant factors that were clinically relevant and associated with faster time-to-regain (defined as p-values ≤ 0.10) were then included as additional potential confounders when assessing the association between time-to-regain and neurodevelopmental outcomes.^21^ Maternal factors significantly associated with time-to-regain were not included as confounders in the final outcomes models as they were all associated with birthweight, as detailed in the Results section (Table 1). Final regression models were adjusted for GA, treatment group, mode of delivery, vasopressor use in the first 14 days (including dopamine, norepinephrine, epinephrine, dobutamine, vasopressin, or milrinone), postnatal steroid use in the first 14 days (hydrocortisone or dexamethasone), birthweight z-score, maternal education, mechanical ventilation at baseline, and quartile of average total fluids in the first 14 days after birth. Relative to a reference quartile, we estimated the adjusted Bayley-III points associated with a given quartile of time-to-regain, birth-to-nadir, nadir-to-regain, maximum weight loss, and cumulative weight loss. For time-to-regain the reference quartile was defined as ≥ 12 days, which is consistent with our hypothesis and also supported via an ancillary study among extremely low birthweight (ELBW) newborns.^5^ For birth-to-nadir, the reference quartile was defined as 3–4 days, which is consistent with our previous publication demonstrating the majority of babies between 24–28 weeks’ gestation reached their weight loss nadir at day 3.^16^ For maximum weight loss, the reference quartile was defined as −5 to −10% maximum weight loss, also consistent with our previous publication’s findings of weight loss of 5–15% being optimal for in-hospital outcomes.^16^ Similarly, when evaluating cumulative weight loss quartiles in adjusted models, −23 to < 0 percent-days was used as the reference group given our previous findings suggesting that some weight loss (5–15%), but not severe weight loss (> 15%), is optimal for EP newborns.^16^ Therefore, we used the quartile that included some, but not severe, weight loss (−23 to < 0) as the reference category.

To determine the time-to-regain components most strongly associated with the outcomes of interest, a manual stepwise forward variable selection approach was used based on the Akaike information criterion (AIC) with a working independence model for multiples. AIC estimates prediction error, with a lower AICs generally indicating a better balance between predictive accuracy and model complexity. For each Bayley-III subscale, the AIC was determined for four separate adjusted models using quartiles of time-to-regain, birth-to-nadir, nadir-to-regain, maximum weight loss, and cumulative weight loss in addition to the covariates above. The model with the lowest AIC was initially selected, and the remaining four time-to-regain components added individually in separate models for the next step. If one of these models had a lower AIC, then that model was selected, and the remaining three variables added in further models. This process continued until adding an additional time-to-regain component did not result in a lower AIC, and the model with the lowest AIC was selected for final inference between the selected time-to-regain components adjusting for other variables as well as the pre-specified covariates. A *p* value < 0.05 was considered statistically significant. All analyses were conducted using the R statistical package (Version 3.6.1, Foundation for Statistical Computing, Vienna, Austria).^22^

## Results

A total of 654 EP newborns enrolled in PENUT had documented weight data to day 14 and survived to receive assessment with at least one Bayley-III subscale. 101 (15.4%) infants did not regain birthweight by day 14 after birth. Quartiles were approximated to the nearest day after birth: ≤5 days (including participants who never lost weight), 6–8 days, 9–11 days, and ≥ 12 days (including those who had not regained birthweight by day 14). For each time-to-regain component, quartiles were similarly generated: birth-to-nadir quartiles: ≤1 day, 2 days, 3–4 days, ≥ 5 days; nadir-to-regain quartiles: ≤1 day, 2–4 days, 5–7 days, and ≥ 8 days; maximum weight loss quartiles: <−15%, −15 to <−10%, −10 to <−5%, and ≥−5%; cumulative weight loss quartiles: ≤−50 percent-days, −50 to <−23 percent-days, −23 to < 0 percent-days, and ≥ 0 percent-days. Table 1 shows the maternal and infant characteristics of the overall cohort and by time-to-regain quartiles. Maternal pre-eclampsia, lower birthweight z-score, increased postnatal steroid and vasopressor exposure, and increased average total fluid administration over the first 14 days after birth were associated with significantly shorter time-to-regain.

### *Association between* time-to-regain *components and 2-Year Neurodevelopmental Outcomes*

**Figure 2:**
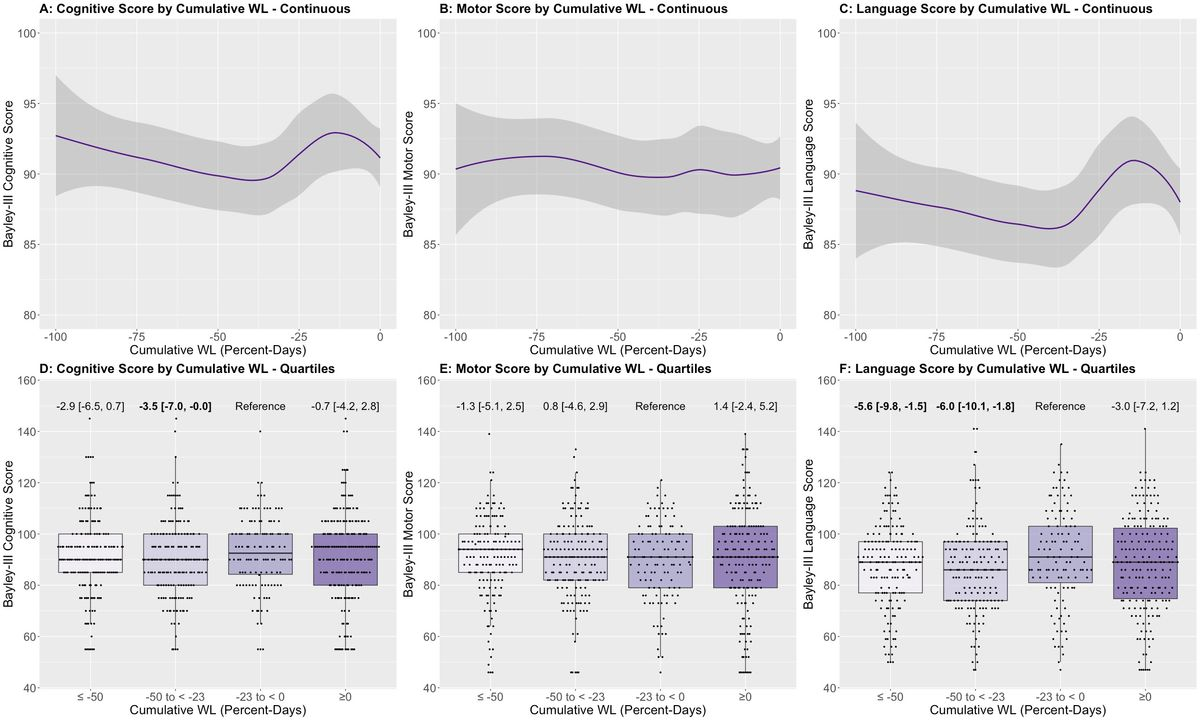
**A-C:** Bayley-III subscale outcomes across weight loss cumulative weight loss (WL), the sum of the percent weight loss for each day the weight was below birthweight). The line represents the local average determined using locally estimated scatterplot smoothing (loess), and the shading represents the 95% confidence intervals. Any infant who took longer than 14 days to regain birthweight was Winsorized to day 15 due to missing weight data from day 14 onwards. **D-F:** Boxplots of Bayley-III cognitive, motor, and language scores by quartiles of weight loss cumulative WL. Adjusted mean difference in subscale score [95% CI] is shown adjusting for gestational age, treatment group, mode of delivery, vasopressor use in the first 14 days (including dopamine, norepinephrine, epinephrine, dobutamine, vasopressin, or milrinone), postnatal steroid use in the first 14 days (hydrocortisone or dexamethasone), birthweight z-score, maternal education, mechanical ventilation at baseline, and quartile of average total fluids in the first 14 days after birth. Significant p-values (<0.05) are bolded.

Qualitatively, time-to-regain was positively associated with cognitive and motor Bayley-III scores (**Supplemental Fig. 1A-C**). When evaluating time-to-regain quartiles in adjusted models, newborns with the longest time-to regain (≥ 12 days) had higher mean Bayley-III cognitive, motor, and language scores compared to the lower three quartiles, but none were statistically significant (**Supplemental Fig. 1D-E**). There were no clear trends or statistically significant associations when evaluating birth-to-nadir, nadir-to-regain, and maximum weight loss with any Bayley-III score at 2-years CA in adjusted models, although nadir-to-regain approached significance with more rapid (≤ 1 day) nadir-to-regain compared to ≥ 8 days (**Supplemantal Figs. 2–4**)

When examined descriptively as a continuous variable, non-linear relationships between cumulative weight loss and Bayley-III scores were seen (Fig. 2A–C). EP newborns with cumulative weight loss − 23 to < 0 percent-days had higher Bayley-III cognitive scores when compared to EP newborns with a greater cumulative weight loss (−50 to <−23: −3.5 points, [95% CI −7.0, −0.0]; Fig. 2D). Similar findings were also seen with Bayley-III language scores (≤−50: −5.6 points, [95% CI −9.8, −1.5]; −50 to <−23: −6.0 points, [95% CI −10.1, −1.8]; Fig. 2F). There was no significant association between cumulative weight loss and Bayley-III motor scores.

## Stepwise selection of multiple weight loss variables

After adjusting for GA, treatment group, vasopressors, steroid use, small for gestational age status (SGA), fluid quartiles, mechanical ventilation, and maternal education, the models including cumulative weight loss quartiles had the lowest AIC for predicting motor and language scores, whereas the nadir-to-regain quartile model had the lowest AIC for predicting motor scores. Adding cumulative weight loss quartiles further lowered the AIC for predicting motor scores, with the addition of nadir-to-regain quartiles lowering the cognitive and language score predictions. The addition of birth-to-nadir, maximum weight loss, and/or time-to-regain quartiles to these models did not further lower AIC. Therefore, the final combined models included both nadir-to-regain and cumulative weight loss to predict all Bayley-III subscales. In the final fully adjusted models (Table 2), significantly lower cognitive scores were seen in those with higher overall cumulative weight loss (≤−50 percent-days: −5.0 Bayley III points, 95% CI −8.9, −1.1 compared with − 23 to 0 percent-days) or those with a rapid nadir-to-regain (≤ 1 day: −5.3 Bayley III points, 95% CI −9.8, −0.8 compared with ≥ 8 days). Also, increasing cumulative weight loss was also associated with lower Bayley-III language scores (≤−50 percent-days: −7.2 Bayley III points, 95% CI −11.8, −2.6 compared with − 23 to 0 percent-days).

## Discussion

This study examined the association between time to regain birthweight and its components including birth-to-nadir, nadir-to-regain, maximum weight loss, and cumulative weight loss with 2-year neurodevelopmental outcomes. While overall time-to-regain quartile was not statistically associated with neurodevelopmental outcomes, a faster nadir-to-regain and increasing cumulative weight loss were associated with lower Bayley-III cognitive scores in the final, AIC, stepwise forward regression model. These associations were more apparent once both cumulative weight loss and nadir-to-regain were included together in the final model, confirming that knowledge of multiple components of weight loss may be required to fully understand their potential impact on outcomes. For instance, nadir-to-regain was not associated with outcomes when examined on its own, but a significant association between rapid nadir-to-regain and adverse neurodevelopmental outcomes was seen once also adjusting for cumulative weight loss. Ultimately, these findings suggest that too rapid a recovery of weight loss (faster nadir-to-regain) or prolonged, excessive cumulative weight loss are potentially detrimental to long-term outcomes of EP newborns. These results suggest a balance of preventing severe, prolonged weight loss while ensuring birthweight is not regained rapidly after the weight loss nadir among EP newborns.

We have not found other publications exploring time-to-regain and its components on long-term outcomes of EP newborns. However, there are several studies that suggest a longer time-to-regain is associated with adverse in-hospital health outcomes among preterm newborns. Specifically, in a single-center, retrospective case-control study in Australia, preterm newborns with type I retinopathy of prematurity had a longer time-to-regain when compared to their matched controls, but these findings did not reach statistical significance (median day of time-to-regain 9 vs 7, adjusted odds ratio 1.08, 95% CI 1.00–1.17, p = 0.059).^6^ The authors concluded that these findings suggest that a longer time-to-regain may aid clinicians in predicting which newborns have retinopathy of prematurity.^6^ Similarly, indicators for malnutrition for preterm infants and neonates were developed by neonatal dietitians who note that longer time-to-regain (> 15 days) among preterm newborns is associated with a diagnosis of malnutrition.^19^ Taken together, current recommendations and published findings suggest a shorter time-to-regain is optimal for preterm newborn outcomes. However, these studies and recommendations do not include evaluation of the rapidity of time-to-regain and its associated components which likely influence overall time-to-regain. Furthermore, these studies do not assess their association with neurodevelopmental outcomes, nor are they specifically focused on the EP population.

Similar to our results, another study reported a longer time-to-regain may be optimal for long-term outcomes among ELBW newborns. Ehrenkranz and colleagues sought to evaluate the association between in-hospital growth velocity and neurodevelopmental outcomes at 18–22 months’ CA among 495 newborns with birthweights between 501–1000 grams.^5^ They divided their cohort into quartiles based upon average in-hospital growth velocities in which growth velocity was defined as “the period between the time that the infant regained birth weight and discharge, transfer, age 120 days, or until a body weight of 2000g was reached.”^5^ The lowest quartile for growth velocity included newborns with a mean weight gain of 12.0 g/kg/day whereas the highest quartile included newborns with a mean weight gain of 21.2 g/kg/day.^5^ Newborns in this highest quartile for growth velocity had significantly less neurodevelopmental impairment compared to the lowest quartile (29% vs 55%, p < 0.001).^5^ Interestingly, newborns in the highest quartile for growth velocity, the group with the least neurodevelopmental impairment, had *longer* time-to-regain than newborns in the lowest quartile (19.5 days vs 15.9 days, p = 0.003).^5^ All 4 quartiles had average time-to-regain *more than* 14 days after birth, supporting that ELBW newborns commonly require more than 2 weeks to regain birthweight.^5^

The current study is novel because it focuses on weight parameters in the first 14 days and their association with neurodevelopment. Most studies that assess the association of neonatal growth velocity on later neurodevelopment outcomes generally either do not include the different components of time-to-regain in the analyses or start *after* birthweight has been regained, thereby overlooking the critical fetal-to-neonatal transition.^5,23,24^ Our study’s findings highlight the need for further exploration into the ideal weight loss patterns among EP newborns with respect to long-term outcomes through prospective, randomized trials.

### Biologic Associations with Time to Regain Birthweight

It is uncertain what constitutes the optimal rate of lean body mass growth velocity during the first 2 weeks after birth among EP newborns. In fact, it is likely that time-to-regain and its components are more a marker of changes in fluid status rather than alterations in lean body mass. During the fetal-to-neonatal transition, EP newborns have increased insensible fluid losses due to their immature organ function including, but not limited to: immature skin barrier, evaporative losses, immature kidney tubular function leading to postnatal diuresis, and respiratory distress.^7–16^ These factors collectively predispose EP newborns to extraordinary fluid and weight loss in the immediate postnatal period. Initial postnatal physiologic weight loss is a necessary, important factor that is associated with improved health outcomes of preterm newborns. This process of physiologic weight loss after birth is critical to a proper fetal-to-neonatal transition, and a lack of weight loss is associated with adverse outcomes including BPD and NEC, among others.^25,16^

Overall, the fetal-to-neonatal transition that occurs over the first weeks after birth is a complex period that involves the interplay between metabolic, endocrine, and fluid alterations. In congruence with other publications demonstrating that minimal or a lack of weight loss after birth is associated with adverse health outcomes,^16,25^ our findings similarly demonstrate adverse neurodevelopmental associations with more rapid time-to-regain (specifically, a more rapid nadir-to-regain) and prolonged, excessive weight loss (increasingly negative cumulative weight loss) among EP newborns.

### Strengths and Limitations

The strengths of our study include the relatively large sample size of 654 EP neonates from 19 academic centers including 30 NICUs across 13 states in the United States.^20^ Due to the multi-center nature of this study, the results may be generalizable to other U.S.-based NICUs and other higher-resourced settings globally. Our study also used a contemporary cohort describing the association between time-to-regain and 2-year neurodevelopmental outcomes among EP infants.

While we attempted to control for variables associated with increased severity of illness such as increased total fluid administration over the first 14 days, postnatal steroid use, postnatal vasopressor use, lower birthweight, and need for mechanical ventilation, it is possible that rapid time-to-regain and associated components are markers of increased severity of illness. Our analyses cannot delineate when or if time-to-regain and associated components reflect fluid management changes versus lean body mass development. Moreover, given that our study occurred in U.S.-based institutions, our findings may not be generalizable to low- and middle-income settings where access to humidification, incubators, intravenous fluids, and other resources may influence both short- and long-term health outcomes. As PENUT only included daily weights through the first 14 days after birth, we are unable to determine the exact timing of regaining birthweight beyond 14 days. This lack of data prevents determination of a potential optimal window for time-to-regain. Finally, our study is a retrospective, secondary analysis of the PENUT trial, and, as such, cannot attribute causality. Future studies targeting specific weight loss ranges or trajectories should be further tested in randomized controlled trials to discern causality prior to implementing these findings in clinical practice.

## Conclusion

Our study highlights that rapid regain of birthweight, especially rapid regain from the weight loss nadir, and prolonged, severe weight loss, are associated with adverse neurodevelopmental outcomes among EP newborns at 2 years CA. Our findings suggest that clinicians caring for EP newborns should consider allowing moderate physiologic weight loss with subsequent gradual time to regain birthweight.

## Figures and Tables

**Table 1:** Maternal and infant demographic, baseline, and exposure variables by time-to-regain quartile. Categorical variables represented by n (%). Continuous variables represented by mean (SD).

		Days from birth to regain birthweight (time-to-regain)				
Maternal or Infant Factor	All Study Participants	≤ 5 days	6–8 days	9–11 days	≥ 12 days	p-value*
Total Number of Infants Surviving to Day 14	654	147	163	155	189	-
**Maternal Factors**						
Maternal Age (years)	29.2 (6.1)	30.2 (5.8)	29.3 (6.5)	29.4 (5.8)	28.3 (6.2)	**0.041**
Multiple Gestation: Yes	148 (22.6)	23 (15.6)	40 (24.5)	38 (24.5)	47 (24.9)	0.44
Chorioamnionitis: Yes	86 (13.1)	13 (8.8)	24 (14.7)	21 (13.5)	28 (14.8)	0.32
Prenatal Steroids						
0 Doses	62 (9.5)	11 (7.5)	15 (9.2)	8 (5.2)	28 (14.8)	**0.019**
1 Dose	129 (19.7)	29 (19.7)	31 (19.0)	26 (16.8)	43 (22.8)	
2 Doses	409 (62.5)	95 (64.6)	100 (61.3)	109 (70.3)	105 (55.6)	
3 + Doses	54 (8.3)	12 (8.2)	17 (10.4)	12 (7.7)	13 (6.9)	
Pregnancy-Induced Hypertension: Yes	49 (7.5)	18 (12.2)	9 (5.5)	12 (7.7)	10 (5.3)	**0.047**
Pre-eclampsia: Yes	92 (14.1)	43 (29.3)	27 (16.6)	13 (8.4)	9 (4.8)	**< 0.0001**
Gestational Diabetes: Yes	38 (5.8)	6 (4.1)	17 (10.4)	7 (4.5)	8 (4.2)	0.15
Cesarean Delivery: Yes	440 (67.3)	112 (76.2)	107 (65.6)	107 (69.0)	114 (60.3)	**0.013**
**Infant Factors**						
Gestational Age (weeks)	25.6 (1.1)	25.4 (1.2)	25.8 (1.1)	24.5 (1.1)	25.6 (1.1)	0.25
Birthweight (g)	815 (189)	685 (176)	824 (166)	837 (174)	891 (178)	**< 0.0001**
5-minute Apgar (median, IQR)	7 (5–8)	7 (5–8)	7 (5–8)	7 (5–8)	7 (5–8)	**< 0.0001**
Sex: Male	335 (51.2)	73 (49.7)	83 (50.9)	82 (52.9)	97 (51.3)	0.99
Birthweight Z-score	−0.1 (0.9)	−0.8 (0.9)	−0.2 (0.8)	0.1 (0.8)	0.3 (0.7)	**< 0.0001**
Intubation/Chest Compressions: Yes	520 (79.5)	124 (84.4)	118 (72.4)	131 (84.5)	147 (77.8)	0.34
**Postnatal Factors in First 2 Weeks**						
Mechanical Ventilation Day 1: Yes	516 (78.9)	132 (89.8)	117 (72.8)	124 (80.0)	143 (75.7)	**0.0047**
Maximum weight loss (%)	−10.7 (6.6)	−4.4 (6.2)	−9.5 (4.5)	−12.3 (4.5)	−15.4 (5.4)	**< 0.0001**
Birth-to-nadir (days)	8.8 (4.4)	2.6 (2.2)	7.2 (0.8)	9.9 (0.8)	14.0 (1.2)	**< 0.0001**
Nadir-to-regain (days)	5.9 (4.7)	1.2 (1.2)	3.7 (1.5)	5.5 (1.9)	11.7 (3.9)	**< 0.0001**
Cumulative weight loss (percent-days)	−30.8 (31.5)	−7.5 (10.7)	−28.3 (19.1)	−52.1 (25.7)	−33.4 (41.0)	**< 0.0001**
Average 14-day Fluid (mL/kg/day)	144 (19)	158 (24)	144 (16)	140 (14)	136 (15)	**< 0.0001**
Treatment Group: Epo	320 (48.9)	69 (46.9)	87 (53.4)	76 (49.0)	88 (46.6)	0.77
Steroids: Yes	110 (16.8)	42 (28.6)	24 (14.7)	20 (12.9)	24 (12.7)	**0.0041**
Vasopressors: Yes	176 (26.9)	57 (38.8)	41 (25.2)	32 (20.6)	46 (24.3)	**0.0099**

* P-value compares differences across weight regain groups using a multivariate Wald test adjusting for gestational age and Epo treatment group.

**Table 2:** Stepwise forward AIC fully-adjusted model demonstrating significant associations between cumulative weight loss and nadir-to-regain with Bayley-III 2-year CA neurodevelopmental outcomes. Adjusted for gestational age, treatment group (Epo or control), vasopressors or steroids use in the first 14 days after birth, birthweight z score, fluid quartiles, maternal education, and mechanical ventilation. Birth-to-nadir, time-to-regain, and maximum weight loss were not selected as predictors in the final model.

	Cognitive Score		Motor Score		Language Score	
Variable/Quartile	Adjusted Bayley-III Points (95%CI)	P-value	Adjusted Bayley-III Points (95%CI)	P-value	Adjusted Bayley-III Points (95%CI)	P-value
Cumulative weight loss ≤ −50 Percent-Days	−5.0 (−8.9, −1.1)	**0.012**	−3.4 (−7.5, 0.8)	0.12	−7.2 (−11.8, −2.6)	**0.002**
Cumulative weight loss −50 to < −23 Percent-Days	−4.6 (−8.1, −1.2)	**0.009**	−2.3 (−6.2, 1.5)	0.24	−7.0 (−11.4, −2.6)	**0.002**
Cumulative weight loss −23 to < 0 Percent-Days	Reference	−	Reference	−	Reference	−
Cumulative weight loss ≥0 Percent-Days	−1.3 (−5.3, 2.8)	0.54	1.5 (−2.8, 5.9)	0.49	−2.7 (−7.3, 2.0)	0.26
Nadir-to-regain ≤ 1 Days	−5.3 (−9.8, −0.8)	**0.022**	−5.1 (−9.8, −0.5)	**0.031**	−4.7 (−9.7, 0.3)	0.065
Nadir-to-regain 2–4 Days	−3.3 (−7.4, 0.8)	0.11	−2.3 (−6.2, 2.0)	0.32	−1.5 (−5.9, 2.8)	0.49
Nadir-to-regain 5–7 Days	0.1 (−3.7, 4.0)	0.95	1.5 (−2.4, 5.4)	0.44	0.7 (−3.5, 4.9)	0.75
Nadir-to-regain ≥ 8 Days	Reference	−	Reference	−	Reference	−

## Data Availability

De-identified individual participant data is available through the NINDSData Archive: https://www-ninds-nih-gov.offcampus.lib.washington.edu/Current-Research/Research-Funded-NINDS/Clinical-Research/Archived-Clinical-Research-Datasets. The data is de-identified and a limited access data set is available through a request form on that page. Data dictionaries, in addition to study protocol, the statistical analysis plan, and the informed consent form will be included. The data will be made available upon publication of all PENUT Trial related manuscripts to researchers who provide a methodologically sound proposal for use in achieving the goals of the approved proposal.

## Abbreviations

AGA: Appropriate for gestational age
BPD: Bronchopulmonary dysplasia
Bayley-III: Bayley Scales for Infant Development 3^rd^ Edition
CA: Corrected ag
CI: Confidence Interval
CWL: Cumulative weight loss
ELBW: Extremely low birthweight
EP: Extremely preterm
EPO: Erythropoietin
GA: Gestational age
GEE: Generalized estimating equations
ICH: Intracranial hemorrhage
IV: Intravenous
NEC: Necrotizing enterocolitis
NICU: Neonatal intensive care unit
OR: Odds ratio
PDA: Patent ductus arteriosus
PENUT: Preterm Erythropoietin Neuroprotection Trial
SGA: Small for gestational age
